# Multiple barriers against successful care provision for depressed patients in general internal medicine in a Japanese rural hospital: a cross-sectional study

**DOI:** 10.1186/1471-244X-10-30

**Published:** 2010-04-26

**Authors:** Tsuyuka Ohtsuki, Masatoshi Inagaki, Yuetsu Oikawa, Akiyoshi Saitoh, Mie Kurosawa, Kumiko Muramatsu, Mitsuhiko Yamada

**Affiliations:** 1 Department of Psychogeriatrics, National Institute of Mental Health, National Center of Neurology and Psychiatry, Kodaira City, Tokyo, Japan; 2 Section of Medical Research for Suicide, Center for Suicide Prevention, National Institute of Mental Health, National Center of Neurology and Psychiatry, Kodaira City, Tokyo, Japan; 3 Oshu City Magokoro Hospital, Oshu City, Iwate, Japan; 4 Iwate Mental Health Center, Morioka City, Iwate, Japan; 5 The Clinical Psychology Course, Graduate School of Niigata Seiryo University, Niigata City, Niigata, Japan

## Abstract

**Background:**

A general internist has an important role in primary care, especially for the elderly in rural areas of Japan. Although effective intervention models for depressed patients in general practice and primary care settings have been developed in the US and UK medical systems, there is little information regarding even the recognition rate and prescription rate of psychotropic medication by general internists in Japan. The present study surveyed these data cross-sectionally in a general internal medicine outpatient clinic of a Japanese rural hospital.

**Methods:**

Patients were consecutively recruited and evaluated for major depressive disorder or any mood disorder using the Patient Health Questionnaire (PHQ). Physicians who were blinded to the results of the PHQ were asked to diagnose whether the patients had any mental disorders, and if so, whether they had mood disorders or not. Data regarding prescription of psychotropic medicines were collected from medical records.

**Results:**

Among 312 patients, 27 (8.7%) and 52 (16.7%) were identified with major depressive disorder and any mood disorder using the PHQ, respectively. Among those with major depressive disorder, 21 (77.8%) were recognized by physicians as having a mental disorder, but only three (11.1%) were diagnosed as having a mood disorder.

Only two patients with major depressive disorder (7.4%) had been prescribed antidepressants. Even among those (n = 15) whom physicians diagnosed with a mood disorder irrespective of the PHQ results, only four (26.7%) were prescribed an antidepressant.

**Conclusions:**

Despite a high prevalence of depression, physicians did not often recognize depression in patients. In addition, most patients who were diagnosed by physicians as having a mood disorder were not prescribed antidepressants. Multiple barriers to providing appropriate care for depressed patients exist, such as recognizing depression, prescribing appropriate medications, and appropriately referring patients to mental health specialists.

## Background

Depression is a common and chronic psychiatric disorder. It is estimated that depression will become the leading cause of disability worldwide in 2030 [1]. In middle-income and high-income countries including Japan, depression was the leading cause of disability in 2004 [1]. Depression is associated with impaired quality of life, yet many depressed patients do not receive appropriate care [2]. The importance of early detection and appropriate care for depressed patients has only recently been recognized.

In the United States and United Kingdom, primary care physicians and general practitioners (GPs) have an important role in diagnosing and treating depressed patients [3,4]. In countries with a primary care system, the importance of developing effective depression management models for primary care settings has been emphasized to provide appropriate care for depressed patients. Collaborative care has emerged as a potentially effective intervention for improving the quality of primary care and patient outcomes, primarily in the US. The effectiveness of collaborative care has been shown in a meta-analysis of US and UK studies [5]. Effective depression management models have been developed and introduced on site in these countries. These models are developed based on situation-specific parameters such as prevalence of depression, recognition rate of depressed patients by physicians, prescription rate of antidepressants to depressed patients, and referral rate to mental health specialists. However, little information necessary for developing effective intervention models is available in Japan.

In Japan, there are few specialists for primary care or general practice because the Japanese medical system has no clear definition regarding the role of primary care and the specific provider responsible. Patients do not need to consult with assigned primary care providers as in the UK medical system. In the Japanese system, patients select hospitals using their own judgment and usually consult general internists, as well as any other specialist, directly. In rural areas, most patients consult a general internist who plays a role similar to that of a primary care physician in the UK. It has been reported that depressed patients in Japanese communities tend to consult not only mental health specialists, but also other specialists such as a general internists because of their somatization in addition to the stigmatization of psychiatric disorders and services [6,7]. The importance of primary care provided by general internists in the management of depressed patients has been stated recently in the Comprehensive Suicide Prevention Initiative published by the Japanese Government. This publication was based on effective intervention models and guidelines for depression care in primary care settings and general practice developed in the US and UK medical systems [8].

A survey examining the prevalence of depression and the recognition rate of depressed patients by physicians was performed nearly 20 years ago. The survey was conducted at general internal medicine outpatient clinics in general hospitals in medium-sized cities of Japan and the patients in the survey were 15-65 years old. The recognition rate of depression by physicians in this survey was lower than in other countries at 19.3% [9]. However, the situation has changed recently as the number of depressed patients receiving medical care has increased [10]. Because of this change in situation, there are no usable data suitable for developing intervention models reflecting the role of primary care in a general internal medicine outpatient clinic in Japanese rural areas.

Meanwhile, the prevalence of chronic medical illness in the elderly is high. Given that a higher prevalence of depression has been reported in patients with chronic medical illnesses [11], general internists have an important role in diagnosing depression among older people, especially in rural areas with a high population aging rate. Also from this perspective, information regarding general internal medicine in rural areas is important.

In the present study, we conducted a survey investigating the prevalence of depression in addition to the ability to recognize depression and rates of psychotropic prescription at a general internal medicine outpatient clinic in a rural hospital. These rates are important indices of each step - diagnosis, judging the care that is necessary, and treating and/or referring the patient to mental health specialists - in the provision of appropriate care for depressed patients by general internists in Japanese rural areas.

## Methods

### Setting

This study was approved by the ethics committee of the National Center of Neurology and Psychiatry in Japan. The researchers provided all participants with detailed information of the study in the form of a written document. The study was performed after obtaining the patients' oral informed consent.

This study was conducted on 6 of 10 consultation days between June 15 and 26, 2009, at a general internal medicine outpatient clinic in a general hospital having no mental health services. This hospital is located in Oshu City, Iwate Prefecture in the Tohoku region of Japan. The hospital is functioning as a regional public hospital and is funded by the National Health Insurance Society at Oshu City. Oshu City is a typical rural area about 500 km north of Tokyo with low influx and efflux of the population. There are high proportions of elderly people and people engaged in primary industry [12].

### Participants

All patients aged 20 or older who visited the outpatient clinic to consult a physician were recruited consecutively. Visitors who consulted for family members or others and patients who had already participated in the survey were excluded. Patients with significant cognitive impairment, those who were unable to understand Japanese, and those who had physical or mental conditions too severe to participate in the survey were excluded. Cognitive impairment was judged by research staff (trained psychiatric nurses, psychiatrists, or trained investigators), based on a semi-structured interview that including asking patients questions such as, "What is the date today?" and "Did you come here by yourself?". The staff sometimes conducted an additional interview regarding the patients' life style and history of dementia if accompanying persons were present.

**Figure 1:**
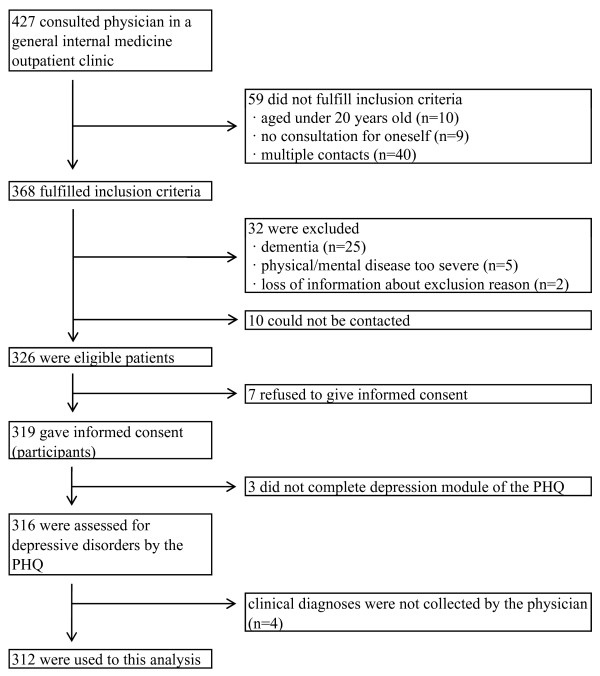
**Sampling Process**.

Figure 1 shows the number of patients included and excluded at each stage of the present study. Of 427 patients who consulted the general internal medicine outpatient clinic during the survey period, 319 patients fulfilled the inclusion criteria and gave informed consent. Three patients had deficits in one or several items of the Patient Health Questionnaire (PHQ: described below) that were needed to evaluate depressive disorders. The questionnaires regarding physician recognition of mental disorders (described below) could not be collected for 4 patients. As a result, we used information from 312 patients in our analyses. The number (%) of patients who could not be contacted, and the number of patients who refused to participate or dropped out from the study were 10 (3.0%) and 14 (4.2%), respectively. The information about sex and age of patients who refused to participate was not collected. Among the seven patients who dropped out from the study, five (71.4%) were female. Age of one patient was unknown, and the mean (standard deviation: SD) age of the six patients was 73.2 (8.4) years.

Five male physicians (mean (SD) age, 44.4 (10.6) years), all of whom had their clinical duties at the outpatient clinic, examined patients at the general internal medicine department in the hospital. Each day, two physicians worked at the routine outpatient clinic in the morning and two others worked there in the afternoon. Each physician saw approximately 15-20 patients, with the four physicians seeing a total of about 60-80 patients in one day.

### Procedure

We approached outpatients visiting the department of general internal medicine during the survey days listed above. Candidate participants who provided informed consent answered several self-report questionnaires during the waiting period for consultation as described in the Measures section below. These questionnaires were used to assess psychiatric disorders, and to survey sociodemographic information and treatment history of mental disorders. Physicians who were blinded to the results of the questionnaires were asked about the diagnosis of primary illness and recognition of mental disorders for each patient after consultation. The history of psychotropic medicine prescription for each patient was collected after the consultation day.

### Measures

#### Clinical diagnosis of primary illness

The clinical diagnosis of primary illness for each patient was made by physicians using a questionnaire that allowed multiple answers and the freedom to provide description.

#### Recognition of mental disorders by physicians

We evaluated the recognition of mental disorders by physicians for each patient using a questionnaire. If any mental disorders were recognized by the physician, a clinical psychiatric diagnosis and the impression of severity were determined by the physician using the following procedure. Clinical psychiatric diagnoses were selected from the following terms: mood disorder, anxiety disorder, alcohol-related disorder, insomnia, dementia, other, and uncategorizable. Multiple selections were allowed. The "other" category included psychiatric disorders or symptoms other than those listed above, and "uncategorizable" indicated that physicians could not clinically diagnose the psychiatric disorder. These terms were determined during a discussion among physicians and researchers prior to the survey period. Because recognition of mental disorders by physicians was intended to reflect clinical diagnoses used daily, not only clinical psychiatric diagnoses but any psychiatric symptoms observed were included as recognition of mental disorders. We defined the severity of mental disorders as the degree of influence on daily life, similar in concept to the Global Assessment of Functioning (GAF) scale in the Diagnostic and Statistical Manual of Mental Disorders, Fourth Edition (DSM-IV) [13]. The physician's judgment concerning the severity of mental disorders was recorded using a 5-point scale ranging from "5 = extremely severe" to "1 = mild," with patients having no mental disorders scored as a zero.

#### Prescription of psychotropic medicine

Data regarding history of psychotropic medicine prescription for all patients on the consultation (survey) day and during the previous 6 months were collected from medical records after the consultation day by two researchers including a psychiatrist (MI and TO).

#### Referral to mental health specialists

History of referral to mental health specialists during the previous 6 months was surveyed from medical records after the consultation day for all patients evaluated as having any mood disorder using the PHQ described below.

#### Depressive disorders and other psychiatric comorbidities

We used the Japanese version of the Patient Health Questionnaire (PHQ) to assess depressive disorders [14]. The PHQ is a self-report version of the Primary Care Evaluation of Mental Disorders (PRIME-MD) [15] that was developed as a primary care screening tool for common mental disorders, including major depressive disorder and probable alcohol abuse or dependence [16,17]. The PHQ has been used in studies all over the world [18,19]. The Japanese PHQ was developed and its validity was assessed using the Mini-International Neuropsychiatric Interview-Plus [14]. We used a 9-item depression module of the Japanese PHQ to assess major depressive disorder and other depressive disorders. Clinical significance of major depressive disorder and other depressive disorders was assessed using a categorical algorithm for the PHQ depressive module. Patients were assessed as having major depressive disorder if they responded "more than half the days" or higher to five or more of the nine items (Questions 1a-1i). Question 1i was included in this total if their response was at least "several days." In addition, the five items had to include either Question 1a or 1b. A patient was considered to have another depressive disorder if they responded with at least "more than half the days" to two, three, or four of the nine items. Again, Question 1i was included in the total items if it received at least "several days", and one of the items had to include either Question 1a or 1b. Patients were considered to have "any mood disorder" when evidence for both major depressive disorder and another depressive disorder was present. The sensitivity and specificity of major depressive disorder were 84% and 95%, respectively [14]. The sensitivity and specificity of any mood disorder were 75% and 94%, respectively (unpublished data analyzed from the data set used in the reference [14]). The severity of depressive disorder was assessed using the summary score (0-27) of each item of the depressive module of the PHQ.

As additional information, we assessed three psychiatric comorbidities: panic disorder, alcohol-related disorder, and generalized anxiety disorder. We used the panic disorder module of the brief PHQ, a simplified version of the PHQ, to assess panic disorder [16]. Although a Japanese version of the brief PHQ has been developed by reverse translation, the validity data have not been reported. We used the probable alcohol abuse or dependence module of the PHQ to assess alcohol-related disorder. The sensitivity and specificity of probable alcohol abuse or dependence in Japanese were 100% and 95%, respectively [14]. We used the Japanese version of the 7-item generalized anxiety disorder scale (GAD-7) to assess generalized anxiety disorder. The GAD-7 is a brief self-report questionnaire used as a screening tool for GAD in clinical practice [20]. Similar to the PHQ, the Japanese version GAD-7 has been developed by reverse translation. Sensitivity and specificity of the Japanese version GAD-7 are 88% and 82%, respectively [21].

### Analysis

We calculated the prevalence and 95% confidence intervals of major depressive disorder and any mood disorder. The recognition rate of mood disorder by physicians and the prescription rate of psychotropic medicine were each calculated as a ratio among patients evaluated as having major depressive disorder and any mood disorder using the PHQ.

We assessed the relationship between the severity of depressive disorder evaluated by the PHQ and the severity of mental disorders based on the physician's judgment using Pearson's correlation coefficient. A two-sided *P*-value < 0.05 was considered significant. We performed statistical analyses using SPSS version 17.0J (SPSS Japan Inc.)

## Results

### Characteristics of the patients who participated in the present study

Among the 312 patients, 193 (61.9%) were female. The median (range) and mean (SD) age were 75 (21-98) and 72.9 (12.5) years. The most common diagnosis of primary illness was hypertension, followed by hyperlipidemia and diabetes (Table 1). Five patients consulted the physician only for mental disorders.

**Table 1:** Clinical diagnosis of primary illness (n = 312).

**Diagnosis**	**n**	**% of patients**
	
Hypertension	165	52.9
Hyperlipidemia	37	11.9
Diabetes	33	10.6
Reflux Esophagitis	17	5.4
Gastritis/Gastric ulcer	14	4.5
Other	132	42.3

Multiple clinical diagnoses were allowed for each patient. The total number of clinical diagnosis for all patients was 398.

Five mental disorders as the primary illness are included in "Other".

The number and prevalence of patients with major depressive disorder and any mood disorder as assessed by the PHQ are shown in Table 2. The number and prevalence of patients diagnosed with panic disorder, alcohol-related disorder, and GAD were 3 (1.0%), 23 (7.4%), and 16 (5.2%), respectively.

**Table 2:** Prevalence of depressive disorders.

	**n**	**%**	**95% CI**
	
Major depressive disorder	27	8.7	5.5-11.8
Any mood disorder	52	16.7	12.5-20.8

Major depressive disorder and any mood disorder, which was defined to include both major depressive disorder and other depressive disorders, were assessed by the PHQ.

CI: confidence interval

The number and prevalence of patients with major depressive disorder comorbid with panic disorder, alcohol-related disorders, and GAD were 2 (7.7%), 1 (4.0%), and 5 (19.2%), respectively. For patients with any mood disorder comorbid with panic disorder, alcohol-related disorders, and GAD, the number and prevalence were 2 (4.0%), 3 (6.4%), and 9 (18.4%), respectively.

### Recognition of mental disorders by physicians

Physicians clinically diagnosed 85 patients as having a mental disorder. The clinical psychiatric diagnoses (number of patients) made by the physicians included the following: mood disorder (15), anxiety disorder (17), alcohol-related disorder (5), insomnia (48), dementia (6), other (13) and uncategorizable (4).

Among the 27 patients identified with major depressive disorder using the PHQ, physicians recognized 21 patients (77.8%) as having a mental disorder. The clinical psychiatric diagnoses made by the physicians for these 21 patients are shown in Table 3. Among the 27 patients with major depressive disorder, only three patients (11.1%) were correctly recognized by physicians as having a mood disorder. Many patients with major depressive disorder were clinically diagnosed with insomnia by physicians.

**Table 3:** Recognition of mental disorders by physicians among patients with major depressive disorder (n = 27) as evaluated by the PHQ.

Recognition by physician	Clinical diagnosis by physician	n	% of patients with major depressive disorder	n	% of patients with major depressive disorder
Any mental disorder		21	77.8		
	Mood disorder			3	11.1
	Anxiety disorder			3^a^	11.1
	Alcohol-related disorder			1	3.7
	Insomnia			14^b^	51.9
	Dementia			1	3.7
	Other			4	14.8
	Uncategorizable			2	7.4
No mental disorder		6	22.2		

Because multiple answers were allowed in the clinical psychiatric diagnosis, the total number of diagnoses was 28 and the number of diagnoses per patient was 1.33 for patients with major depressive disorder. Also, the numbers for anxiety and insomnia include patients diagnosed with a mood disorder: ^a^1, ^b^2.

Meanwhile, among the 52 patients diagnosed with any mood disorder using the PHQ, physicians recognized 31 patients (59.6%) as having a mental disorder. The clinical psychiatric diagnoses made by the physicians for these 31 patients are shown in Table 4. Among the 52 patients with any mood disorder, physicians recognized only seven patients (13.5%) as having a mood disorder.

**Table 4:** Recognition of mental disorders by physicians among patients with any mood disorder (n = 52) as evaluated by the PHQ

Recognition by physician	Clinical diagnosis by physician	n	% of patients with any mood disorder	n	% of patients with any mood disorder
Any mental disorder		31	59.6		
	Mood disorder			7	13.5
	Anxiety disorder			5^a^	9.6
	Alcohol-related disorder			2	3.8
	Insomnia			18^a^	34.6
	Dementia			1	1.9
	Other			6	11.5
	Uncategorizable			2	3.8
No mental disorder		21	40.4		

Because multiple answers were allowed in the clinical psychiatric diagnosis, the total number of diagnoses was 41 and the number of diagnoses per patient was 1.32 for patients with any mood disorder. Also, the numbers for anxiety and insomnia include patients diagnosed with a mood disorder: ^a^3.

Among the 85 patients who were recognized by physicians as having a mental disorder, the physicians judged the severity of the mental disorders (number of patients) as follows: extremely severe (1), moderately severe (7), moderate (20), moderately mild (30), or mild (24). The severity scores for three patients were blank.

Among patients identified with any mood disorder using the PHQ, the relationship between depression severity using the PHQ summary score and the severity of the mental disorder as judged by the physician was significant (Pearson's correlation coefficient r = 0.346, p = 0.012). Among the 27 patients with major depressive disorder, 12 patients had moderately severe depression (summary score of the PHQ: 15-19) or severe depression (20-27). Among these, physicians judged seven patients (58.3%) as having a moderately mild or a mild mental disorder, or no mental disorders. In short, physicians underestimated the severity of their disorders.

### Prescription of psychotropic medicine by physicians

The survey of psychotropic prescription history showed that 13 (4.2%) patients were prescribed any antidepressant including sulpiride, which is permitted by insurance as a drug for depression in the Japanese health system, and 72 (23.1%) were prescribed an anxiolytic or hypnotic. Two patients had been prescribed an antiepileptic. The numbers (%) of psychotropic medicine prescriptions in patients identified with major depressive disorder and any mood disorder using the PHQ are shown in Table 5. Among the 27 patients with major depressive disorder, only one patient had been prescribed an antidepressant by a physician and another patient was prescribed an antidepressant by another outpatient clinic (orthopedic department) in the same hospital. In addition to the two patients prescribed antidepressants by physicians, one patient had been prescribed an antidepressant from another hospital. As a result, only three patients with major depressive disorder had received any antidepressants.

**Table 5:** Prescription of psychotropic medicine by physicians.

	**Major depressive disorder** **n = 27**		**Any mood disorder** **n = 52**	
	
	**n**	**%**	**n**	**%**
	
Antidepressant (including sulpiride)	2	7.4	5	9.6
Anxiolytic/Hypnotic	16	59.3	22	42.3
No psychotropic medicine	11	40.7	29	55.8

% is in patients with depressive disorder evaluated by the PHQ.

Patients prescribed both antidepressant and anxiolytic/hypnotic: Major depressive disorder (2), any mood disorder (4).

One patient with major depressive disorder who was prescribed an antidepressant from another hospital was not included.

Even among those who were clinically diagnosed as having mood disorders by physicians irrespective of the PHQ depression score (n = 15: three with major depressive disorder, four with other depressive disorder, and eight without any mood disorder), only four (26.7%) were prescribed an antidepressant.

Additionally, according to medical records, none of the patients identified with any mood disorder using the PHQ had been referred to a mental health specialist.

## Discussion

PHQ results from patients visiting a general internal medicine outpatient clinic of a rural hospital showed that the prevalence of major depressive disorder and any mood disorder were 8.7% and 16.7%, respectively, in this population. However, among the patients with major depressive disorder, the physician recognition rate of mood disorder was 11.1%. The prescription rate of antidepressants to patients with major depressive disorder was 7.4%. Even in patients who were clinically diagnosed by physicians as having a mood disorder, the prescription rate of antidepressants was only 26.7%.

### Prevalence

In a survey performed nearly 20 years ago using the Composite International Diagnostic Interview (CIDI) at general internal medicine outpatient clinics in Japanese general hospitals, the prevalence of depression was 3.0% [9]. The prevalence of major depressive disorder in the present study was higher than that in the previous study. The previous survey included patients 15-65 years old, while most of the participants in this study were older (mean age: 72.9 years old). In addition, the study sites of the previous survey were located in medium-sized cities in Nagasaki Prefecture, but the present study was performed in a rural hospital. These differences in patient characteristics and hospital settings may partly explain the higher prevalence of depression in the present study.

A meta-analysis of several studies in other countries showed that the prevalence of depression in primary care settings for people aged 65 or older is 15.9% [22]. The prevalence of major depressive disorder in this study was 8.7%, lower than in other countries. This may be partially due to a difference in medical systems because patients can directly consult mental health specialists in Japan rather than being required to consult primary care physicians, as is common in other countries. Meanwhile, in a previous epidemiological study of people in a Japanese community, the 12-month prevalence of major depressive disorder was 2.9% [23]. The lower prevalence in the community may be reflective of the lower prevalence of depression diagnosed in a general internal medicine outpatient clinic. Although a direct comparison is limited by differences in response rate, age distribution, and survey method, the prevalence of depression in a general internal medicine outpatient clinic of a rural hospital in the present study was higher than the prevalence in the community. This is consistent with results reported from the US and UK showing the prevalence of depression in primary care settings is higher than in the community [22,24]. This means that depressed patients who have not received appropriate treatment have consulted general internists in spite of Japan's medical system that allows direct consultation to specialists. It is important that physicians appropriately recognize depressed patients and treat and/or refer them to mental health specialists. These physicians can play a role in gatekeeping unrecognized and untreated depressed patients to provide them with appropriate care.

### Recognition

The recognition rate (11.1%) of major depressive disorder in the present study was lower than the rate of depression reported in the previous Japanese study (19.3%) [9]. Hospitals in the previous study had their own psychiatric units, and thus physicians in those hospitals may have frequently examined patients with psychiatric disorders and become proficient in diagnosing depression. However, the hospital in the present study did not have a psychiatry department and no mental health services were provided by mental health specialists. Despite this difference between the Japanese studies, both recognition rates in Japan were much lower than those in other countries as shown by a meta-analysis (47.3%) [22]. Therefore, as a first step, it is necessary to increase the recognition rate of depressed patients by physicians in Japan. Effective screening of depression [18,19] may be a key activity for improving depression care.

A simulation in the meta-analysis suggested that when the prevalence is 10%, there are more false positives (n = 16.8) than either missed (n = 5) or identified cases (n = 5) for every 100 unselected cases seen in primary care. There was concern that false positives would increase as the prevalence decreased [22]. In the present study, not only the physician recognition rate of depressed patients was low, but also the false positive rate of was low (3.1%). This may mean that physicians do not pay attention to depressive disorder. General internists may think that care of depression is not "their business" in the Japanese medical system and that depressed patients should directly consult mental health specialists. To introduce an effective screening system, education to increase awareness and to change physician attitudes toward depression may be important.

Although the severity of mental disorders judged by physicians correlated with the severity of depression assessed by the PHQ (Pearson's correlation coefficient r = 0.346, p = 0.012), more than half of the patients with severe depression were misjudged as having depression of mild to moderate severity, or having no mental disorder (58.3%). This result suggests that appropriate care for depression was not provided even to severely depressed patients who really needed care. In addition to constructing and implementing a system of screening for depression, a referral system to mental health specialists and/or an increase in physician diagnostic and treatment skills is needed.

Many patients identified with major depressive disorder using the PHQ were recognized as having a mental disorder by physicians, but physicians often clinically diagnosed the disorder as insomnia, which is a common symptom of depressive disorders. The higher physician recognition rate of any mental disorder, such as insomnia, may be useful in prompting the suspicion of depression. When a physician notes insomnia and/or a mental disorder in a patient, they should at least screen for depression using a validated screening tool. This step will increase the recognition rate of probable depression by physicians.

Of patients with major depressive disorder, only two were prescribed antidepressants and many were prescribed anxiolytics or hypnotics. This may be creating a further significant problem of likely dependence on the medication. In addition, no patients were referred to mental health specialists. These results seem consistent with the higher rate of insomnia clinically diagnosed by physicians, the lower rate of correct clinical diagnosis of depression, and the lower estimate of the severity of mental disorders. Even for patients judged by physicians as having a mood disorder, the prescription rate of antidepressants by physicians was low (26.7%). Although it is controversial whether antidepressants should be prescribed to patients with mild depression in primary care settings [3,25], the results of the present study suggest that appropriate care may not always be provided for depressed patients even when physicians become able to accurately diagnose depression. Given such a situation, physicians must at least recognize and monitor depressive disorders to judge the necessity of care and referral to mental health specialists.

### Advantages of the study

No prior study has surveyed recent data of depression prevalence and physicians' recognition rate of depression at a general internal medicine outpatient clinic in Japan. In addition, this is the first study reporting prescription rates of antidepressants to all consulted patients.

The present study was performed in a hospital located in a rural area where the proportion of the elderly is high. Generally, medical resources are poorer in rural areas than in urban areas, and elderly people have more chronic physical illnesses. Thus, general internal medicine in a rural area has an important primary care role in the community, especially for the elderly. In fact, most participants in the present study were geriatric patients. The findings are useful for constructing an effective intervention model to care for depressed patients in rural areas in Japan.

The rate of patients who did not participate in a similar survey performed in a rural French area using the PHQ was 14.1% (11.4% refused to participate, and 2.7% did not have enough to time to answer) [26]. The rate of patients who did not participate in the present study was half (7.1%) that of the French study. This suggests that the bias caused by refusal to participate in the present study may be smaller than that of the previous study. Furthermore, the rate of patients who did not participate in the survey using the Structured Clinical Interview for DSM-IV (SCID) was more than 40% [27]. Use of the PHQ instead of a semi-structured interview is one reason for the increased rate of participants. However, the bias from using the PHQ, which is a self-administered questionnaire, instead of a semi-structured interview may be unavoidable, as discussed in the following section.

### Limitations of the study

The present study has several limitations. First, as discussed above, we used self-administered questionnaires (the PHQ and the GAD-7) to evaluate depressive disorders and comorbid psychiatric disorders. The PHQ addresses symptoms only for a two-week period and may include bereavement reactions, mood disorders caused by physical disorders or medications, and/or depressive episodes of bipolar disorders. Although the Japanese PHQ has high sensitivity and specificity for major depressive disorder, evaluation using a diagnostic interview, such as the semi-structured clinical interview for DSM-IV, will increase the validity of the results. Second, we surveyed only five physicians in one hospital. To increase the generalizability of the present results, a study including multiple hospitals or clinics is needed. Third, we judged cognitive impairment based on brief semi-structured interviews of patients or accompanying persons. Sometimes it is difficult to discriminate between depression and cognitive impairments caused by dementia in the geriatric population. A study using a screening or diagnostic tool with higher performance to exclude cognitive impairment is needed. Finally, we surveyed a history of psychotropic medicine prescription on the consultation day. However, the prescription may be reflected behavior by previous physicians rather than the one carrying out the current diagnosis.

## Conclusions

The prevalence of depression at a general internal medicine outpatient clinic was higher in the present study than in the Japanese community. Thus, general internists can play a role as gatekeepers for diagnosing untreated depressed patients in the community. However, physicians did not recognize depressed patients, even in severe cases. The prescription rate of antidepressants to depressed patients and the referral rate of depressed patients to mental health specialists were also low. In addition, the prescription rate of antidepressants to patients whom physicians diagnosed as having a mood disorder was also low.

There are multiple barriers to providing appropriate care for patients with depression, such as recognition of depression, judgment of its severity, prescription of antidepressants and referral to mental health specialists. Collaborative care models developed and shown to be effective in the US and UK [5] to care for depressed patients by general practitioners and primary care physicians cannot be applied directly to the Japanese medical system.

Physicians can recognize insomnia comorbid with depression and can judge the presence of a mental disorder in depressed patients. Thus, an important step is to change physicians' attitude to depression into "it is our business" to find depression. The additional step is to perform screening and then to monitor the screening-positive patients and to refer them to mental health specialists. In addition to constructing a screening and monitoring system of depression, an educational intervention for physicians is key for improving the quality of life of depressed patients at general internal medicine outpatient clinics and of missed depressed patients in the community.

## Competing interests

MI received speaking fees from Eli Lilly.

## Authors' contributions

All authors have read and approved the final version of the manuscript.

MI was the principal investigator and developed the original idea for the study. TO, MI, YO, and MK designed the study. TO, MI, YO, MK, and AS performed the survey. KM developed several Japanese questionnaires used in our survey. TO and MI analyzed data and prepared the manuscript. MY was a supervisor.

## Pre-publication history

The pre-publication history for this paper can be accessed here:

http://www.biomedcentral.com/1471-244X/10/30/prepub
